# Supplementary material for: Complexome profiling of the Chlamydomonas psb28 mutant reveals TEF5 as an early PSII assembly factor
Source: Plant Cell. 2025 Mar 17;37(6):koaf055. doi: 10.1093/plcell/koaf055 (PMC12164590; doi:10.1093/plcell/koaf055)
Supplement: koaf055_Supplementary_Data [file koaf055_supplementary_data.zip › psb28_interactive.zip!/plots/intensity/qualityPlots/peptides/_Cre07.g353230.t1.2.html]

### Cre07.g353230.t1.2

Trivial name:   
SeqCoverage [median]: 6.50  
Mass: 51.35 kDa  
  
 

| Sequence | MissedCleavage | Mass [Da] | wtIntensity | muIntensity | Score |
| --- | --- | --- | --- | --- | --- |
| ALAAVELADGL | 0 | 1041.57 | 321.20 | 0.00 | 136.16 |
| EAVVQAGR | 0 | 828.45 | 1046.99 | 440.51 | 134.06 |
| GVEPAHLLGALTR | 0 | 1332.75 | 861.01 | 364.75 | 62.04 |
|  |  |  | 2229.2 | 805.3 |  |
